# Effective stakeholder engagement: design and implementation of a clinical trial (SWOG S1415CD) to improve cancer care

**DOI:** 10.1186/s12874-019-0764-2

**Published:** 2019-06-11

**Authors:** Sarah Barger, Sean D. Sullivan, Ari Bell-Brown, Brad Bott, Anne Marie Ciccarella, John Golenski, Mark Gorman, Judy Johnson, Karma Kreizenbeck, Florence Kurttila, Ginny Mason, Jamie Myers, Carole Seigel, James L. Wade, Guneet Walia, Kate Watabayashi, Gary H. Lyman, Scott D. Ramsey

**Affiliations:** 1 Hutchinson Institute for Cancer Outcomes Research, Seattle, WA USA; 2 CHOICE Institute, School of Pharmacy, University of Washington, Seattle, WA 98195 USA; 3 Intermountain Healthcare, Salt Lake City, UT USA; 4 Independent Patient Research Partner and SWOG Digital Engagement Committee Member, New York, NY USA; 5 Kairoi Healthcare Strategies, San Francisco, CA USA; 6 Cancer Survivor Advisor, Silver Spring, MD USA; 7 SWOG Lung Committee Patient Advocate, St. Louis, MO USA; 8 SWOG GI Committee Patient Advocate, Citrus Heights, CA USA; 9 SWOG Breast Committee Patient Advocate, West Lafayette, IN USA; 10 University of Kansas, School of Nursing, Kansas City, KS USA; 11 SWOG GI (Pancreatic Cancer) Committee, Patient Advocate, Boston, MA USA; 12 Heartland NCORP, Decatur, IL USA; 13 Genentech, Inc, South San Francisco, CA USA

**Keywords:** Stakeholder engagement, Patient engagement, Oncology, Clinical trial, Cancer, Cancer care delivery research

## Abstract

**Background:**

The Fred Hutchinson Cancer Research Center has engaged an External Stakeholder Advisory Group (ESAG) in the planning and implementation of the TrACER Study (S1415CD), a five-year pragmatic clinical trial assessing the effectiveness of a guideline-based colony stimulating factor standing order intervention. The trial is being conducted by SWOG through the National Cancer Institute Community Oncology Research Program in 45 clinics. The ESAG includes ten patient partners, two payers, two pharmacists, two guideline experts, four providers and one medical ethicist. This manuscript describes the ESAG’s role and impact on the trial.

**Methods:**

During early trial development, the research team assembled the ESAG to inform plans for each phase of the trial. ESAG members provide feedback and engage in problem solving to improve trial implementation. Each year, members participate in one in-person meeting, web conferences and targeted email discussion. Additionally, they complete a survey that assesses their satisfaction with communication and collaboration. The research team collected and reviewed stakeholder input from 2014 to 2018 for impact on the trial.

**Results:**

The ESAG has informed trial design, implementation and dissemination planning. The group advised the trial’s endpoints, regimen list and development of cohort and usual care arms. Based on ESAG input, the research team enhanced patient surveys and added pharmacy-related questions to the component application to assess order entry systems. ESAG patient partners collaborated with the research team to develop a patient brochure and study summary for clinic staff. In addition to identifying recruitment strategies and patient-oriented platforms for publicly sharing results, ESAG members participated as co-authors on this manuscript and a conference poster presentation highlighting stakeholder influence on the trial. The annual satisfaction survey results suggest that ESAG members were satisfied with the methods, frequency and target areas of their engagement in the trial during project years 1–3.

**Conclusions:**

Diverse stakeholder engagement has been essential in optimizing the design, implementation and planned dissemination of the TrACER Study. The lessons described in the manuscript may assist others to effectively partner with stakeholders on clinical research.

## Background

Recognition of the value and importance of integrating stakeholder engagement into comparative effectiveness research (CER) and patient-centered outcomes research (PCOR) to improve health care delivery continues to grow [1, 2]. Stakeholder engagement in research is defined as an iterative process of actively soliciting the knowledge, experience, judgment and values of individuals selected to represent a broad range of interests in a particular issue, for the dual purposes of creating a shared understanding and making relevant, transparent and effective decisions [1]. Patients and other relevant stakeholders can support the objectives of CER and PCOR by contributing to evidence-based, patient-centered health interventions and tools that help patients and consumers make informed health care decisions [3]. Studies have found that engaging stakeholders in the development and execution of CER studies influenced research prioritization [4–6], provided valuable insights regarding study design [7, 8] and data analysis [9], and led to greater relevance and uptake of research results [10, 11]. In particular, patients may enhance CER and PCOR by providing experiential knowledge to a research question or study design, informing data collection and analysis, reviewing and interpreting results, and improving translation and dissemination of key findings [12–14].

Despite the potential benefits, there are significant challenges in embedding stakeholders into the research continuum. Consistent engagement requires a significant investment of time and resources from both researchers and stakeholders [13, 15]. Deverka and colleagues evaluated stakeholder perceptions of engagement in CER and identified barriers including unclear role expectations, limited opportunities to communicate with clinical investigators, and difficulty in balancing timely input from stakeholders with the pace of research needs [16]. At present, no general agreement exists regarding best practices of longitudinal stakeholder engagement in CER [1, 10]. In oncology, there is experience with multi-stakeholder engagement in CER trial design [17–19] but no experience with ongoing engagement from design through trial implementation and dissemination. Accordingly, the purpose of this manuscript is to describe one model for longitudinal stakeholder engagement that has been effectively implemented to support a pragmatic clinical trial in cancer care delivery.

Known as TrACER (A Pragmatic Trial Assessing CSF Prescribing Effectiveness and Risk), this randomized, controlled CER study evaluates a guideline-based, automated order entry system for prescribing primary prophylactic colony stimulating factors (CSF) against usual care (i.e. physician-directed CSF prescribing) for patients receiving myelosuppressive chemotherapy [17]. TrACER is one of 23 studies in the PCORI Pragmatic Clinical Studies Program [20] and is being conducted by SWOG through the National Cancer Institute Community Oncology Research Program (NCORP) network in 45 clinics that support cancer care delivery research. The primary goals of the trial are to determine the impact of the order entry system on CSF prescribing, overall rates of febrile neutropenia (FN) and the rate of FN among patients receiving intermediate risk chemotherapy. Secondary objectives focus on the impact of the intervention on patient-centered outcomes, such as health-related quality of life and knowledge of the benefits and risks of CSF. The TrACER Study attempts to answer questions that are important to patients and healthcare systems and therefore involves a variety of stakeholders in the design, implementation and dissemination of results.

This manuscript describes the process and resultant impact of engaging a large group of stakeholders in the planning and implementation of the TrACER Study. Our approach and lessons learned contribute to the growing evidence of the value of stakeholder engagement in CER, specifically in oncology.

## Methods

### Initial stakeholder engagement in TrACER

Prior to submitting a proposal for funding, the research team assembled an External Stakeholder Advisory Group (ESAG) to inform each phase of the trial from planning and design to implementation and dissemination. The ESAG is comprised of 21 national leaders from 14 different states and territories in the United States, including ten patient partners, two payers, two pharmacists, two guideline experts, four providers (nursing, medical oncology, internal medicine) and one medical ethicist. Four patient partners joined the ESAG during the proposal planning phase, and six additional patient partners from the SWOG Patient Advocate Committee [21] joined the group one year into the trial. All patient partners had prior experiences working with cancer care professionals and/or researchers and navigating the health care system in their roles as patient advocates, research partners, patients/survivors and caregivers. In alignment with PCORI’s compensation framework [22], patient partners received financial compensation for their time and effort as study advisors and were reimbursed for all costs related to travel to ESAG meetings.

### Development of engagement plan

**Fig. 1:**
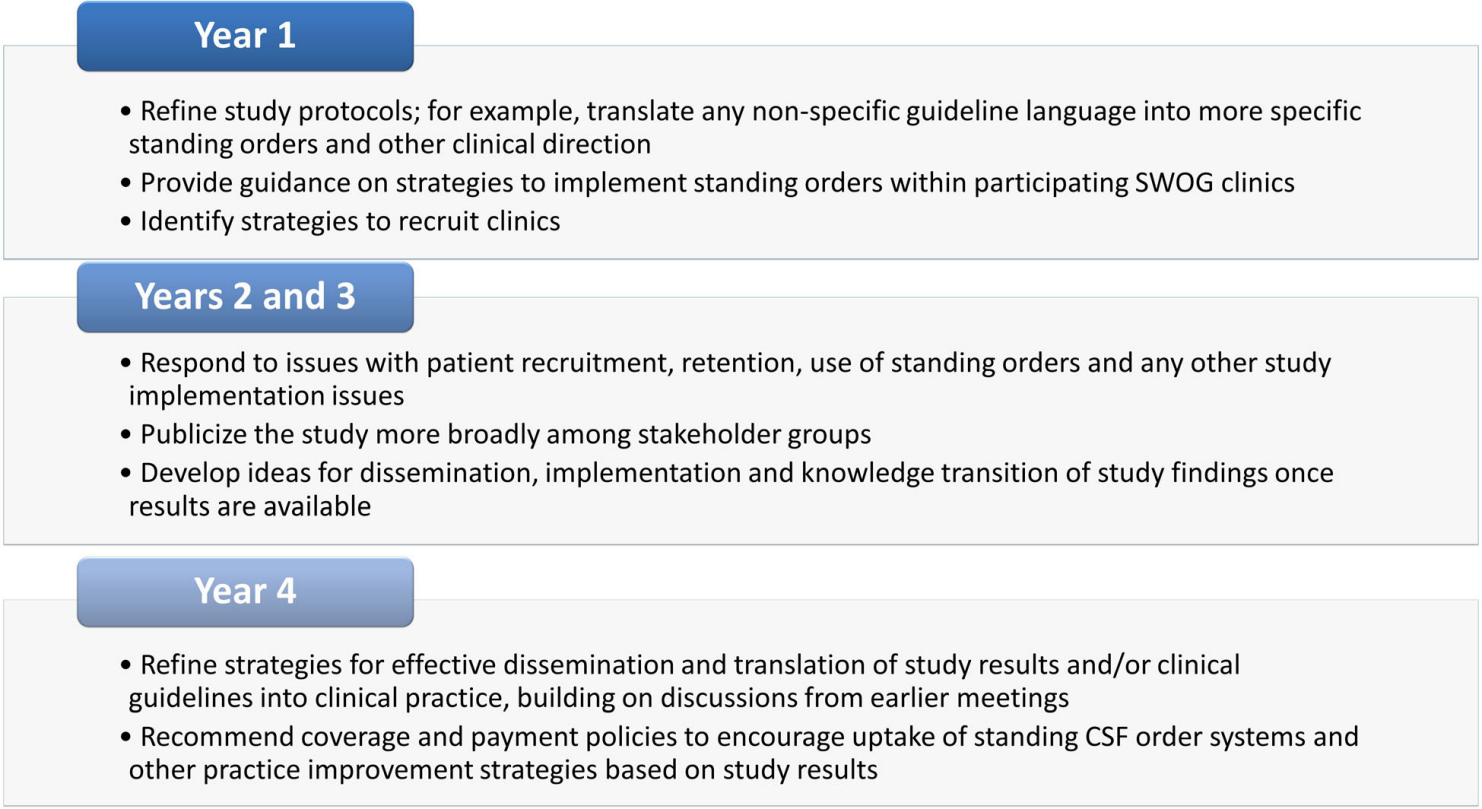
ESAG Activities According to Engagement Plan (H: 3.53″, W: 6.39″)

During the planning phase, the research team developed a four-year engagement plan outlining the aims of stakeholder engagement, roles and responsibilities of ESAG members and their expected activities throughout the trial. As demonstrated in Fig. 1, stakeholders are embedded in the lifecycle of the clinical trial, from design and protocol development through interpretation and dissemination of results.

### Structure and facilitation of ESAG meetings and communication

Each year, the stakeholders and research team convene during two web conferences, two patient partner-specific web conferences and one in-person meeting held in conjunction with the SWOG Group Meeting. These interactive meetings facilitate regular communication about trial progress, open discussion and collaborative problem solving. During patient-specific meetings, the research team provides a study briefing with an explanation of any relevant clinical or statistical concepts that may be unfamiliar; in addition, the group focuses on patient-specific topics like barriers to patient recruitment. The team reaches out to specific stakeholders over email to request feedback on issues related to their areas of expertise. For example, guideline experts and pharmacists on the ESAG provided input over email during the review of the FN risk algorithm in 2015 and 2017 to ensure that the algorithm reflects up-to-date guidelines from the National Comprehensive Cancer Network (NCCN). To ensure timely decision-making regarding study issues, stakeholders offer their recommendations during a two-week comment period. The research team discusses feasibility of ESAG suggestions, determines a course of action, and reports back to group on the final decisions made.

### Annual stakeholder evaluation and reporting

The research team evaluates stakeholder engagement, satisfaction and impact on the trial annually through the ESAG Feedback Survey. The 21-question survey addresses communication, meeting structure, involvement and input, respect and value, and ESAG areas of interest for the next project year. Comment boxes provide an opportunity for additional feedback or suggestions. Stakeholders complete the survey prior to the annual in-person ESAG meeting so that the research team can share the results with the ESAG and discuss the next year’s engagement activities.

## Results

**Fig. 2:**
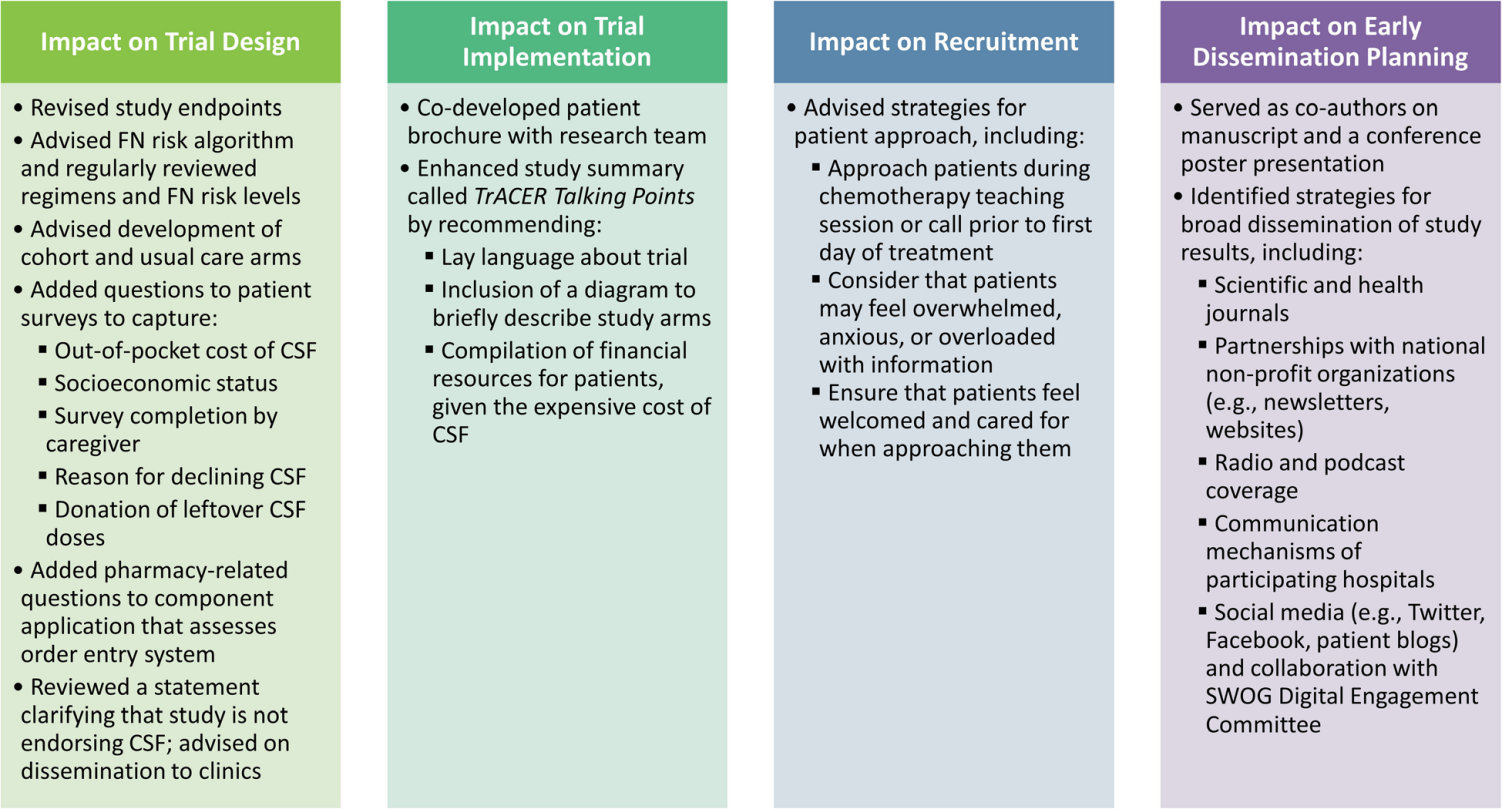
Summary of ESAG Impact on Trial (H: 4.13″, W: 6.5″)

The research team solicits and integrates ESAG feedback into the trial during each phase. Below and in Fig. 2, we describe how ESAG feedback has impacted trial design, implementation, recruitment and early dissemination planning.

### Impact on trial design

#### Proposal planning

During the proposal planning phase, the ESAG advised the research team to adjust trial endpoints and base the standing order intervention solely on chemotherapy regimen risk, not individual patient characteristics such as comorbidities. In addition, the group raised concerns about only consenting patients in the intervention arms, so the research team extended the consent process to participants in all arms. ESAG members recommended that the research team track oral antibiotic use and clarify that physicians can override standing orders and prescribe CSF based on clinical judgment. These suggestions were incorporated into the protocol.

#### Site feasibility survey

When giving feedback on the trial design, the ESAG recognized the importance of measuring and characterizing usual care, given the heterogeneity of existing prescribing patterns. To better understand CSF prescribing patterns, the research team decided to survey clinic practices, conduct a retrospective audit of each practice’s patterns for the 6 months prior to the trial, and add a parallel observational cohort study of practices ineligible for the primary randomization. In addition, the ESAG and research team developed a site feasibility survey to help assess clinic eligibility for the trial and understand practice-level characteristics as they pertain to prescribing CSF.

#### Patient surveys

ESAG patient partners made several recommendations for improving the trial’s baseline and follow-up patient surveys. They emphasized the importance of tracking cost of CSF, which led the research team to add a question capturing out-of-pocket costs of CSF. In addition, they noted that patients sometimes receive CSF “donations” from other patients due to its high cost, so a question was added to capture if patients had any remaining doses of CSF, how much they had, and what they did with the remaining doses. Questions also were added to capture socioeconomic status (i.e. annual household income), who completed the survey (including a caregiver option) and the patient’s understanding of his/her risk of febrile neutropenia.

#### Protocol and consent

The ESAG refined numerous protocol areas, including site recruitment, data collection, trial design and randomization, order entry intervention and criteria for evaluation and endpoint analysis. Nurses and patient partners provided several suggestions to improve clarity of the consent forms for participants.

#### FN risk algorithm

A febrile neutropenia (FN) regimen-based risk algorithm based on current NCCN guidelines and peer-reviewed studies was developed by the research team with feedback from pharmacy and guideline experts in the ESAG. Each year when new NCCN guidelines are released, this risk algorithm is reviewed and updated by the same stakeholders.

### Impact on trial implementation

#### TrACER patient brochures

The ESAG suggested that it would be helpful to give patients a handout explaining the trial in lay terms. During an eight-month process, the patient partners collaborated with the research team to create a trifold brochure in both English and Spanish that describes the trial in a clear, understandable manner. Patient partners assisted in replacing medical jargon with non-technical language and articulating the expectations of patient participation, as well as risks and benefits. Study site staff reported that the brochure was highly valued as a simple tool for explaining the trial to eligible patients and a helpful summary document for clinic staff and providers.

#### TrACER talking points

In August 2016, study coordinators at participating sites recommended creating a summary handout to assist in answering any patient questions about the trial. In collaboration with ESAG patient partners, clinic coordinators, and the TrACER SWOG data coordinators, the research team developed *TrACER Talking Points*. This document includes a trial overview for investigators and patients, as well as a clinic group diagram with a brief overview of the different study arms.

#### Physician statement

Based on physician concerns regarding “mandated” CSF use, the research team developed a statement to clarify that the protocol-indicated standing orders should not be viewed as a clinical endorsement for the inclusion or exclusion of CSF in the chemotherapy order. Physicians on the ESAG reviewed this language and suggested that the team disseminate a shorter statement via the study newsletter and a longer statement for the study website, which was implemented accordingly.

### Impact on recruitment

#### Patient approach, recruitment and consent

The TrACER Study opened to accrual in October 2016. The patient partner and ESAG web conferences in Spring 2017 focused on barriers to patient accrual, specifically the patient approach. To participate in the trial, patients must be consented prior to their first cycle of chemotherapy. Thus, ESAG members compiled a list of strategies for approaching patients during this narrow timeframe, and this information was shared with sites that struggled to accrue patients. ESAG feedback also was incorporated into the trial’s frequently asked questions document available for all sites via the SWOG/Cancer Trials Support Unit study website.

### Impact on dissemination and future directions

From the beginning of the trial, the ESAG has been vocal about the importance of disseminating results to all stakeholders using multiple mediums. During one web conference, the group identified various methods for dissemination of trial results, including social media (i.e. patient blogs, Facebook, Twitter), publications such as CURE magazine, newsletters of national advocacy organizations and radio talk shows. They encouraged the research team to incorporate results into pertinent guidelines (e.g., NCCN, American Society of Clinical Oncology) and to publish findings, both positive and unsuccessful results, in open access academic journals for the learning value. In addition, patient partners emphasized the importance of conveying results to participants and their caregivers/families and provided insights into appropriate vehicles for achieving this communication that will not burden clinical trial investigators.

During project year 3, the patient partners of the ESAG served as co-authors on a poster detailing the influence of patient engagement on trial design and implementation that was presented at a national quality conference. Importantly, ESAG members contributed to the development of this manuscript’s structure and content through planning calls and email communication over the course of one year. During year 4, the ESAG will continue advising the study team on patient enrollment and dissemination planning. In addition, ESAG members will review and provide feedback on reports from the Data and Safety Monitoring Committee, which include recommendations for changing the study based on interim analyses.

## Discussion

ESAG members have played a significant role in optimizing the design, implementation, recruitment, and early dissemination planning of the TrACER Study. The group’s diverse perspectives and experiences have enriched group discussion about the trial and catalyzed real-time solutions to challenges over the course of three years. For example, patient partners have drawn on their varied experiences as clinical trial participants, chemotherapy patients, study coordinators, caregivers and patient advocates to contextualize challenges around patient recruitment and propose effective strategies to overcome these challenges. Guideline experts, providers and pharmacists have contributed invaluable clinical knowledge about CSF prescribing and ensured that the research team is responsive to frequently changing guidelines. Our medical ethicist, payers and patient partners have initiated important discussions about the high cost of CSF and the resultant impact on patients, particularly those involved in the trial. In sum, the continuous and strategic involvement of ESAG members in the trial facilitated critical thinking, sharing of unique insights, and collaborative problem solving, which led to key improvements in trial design and implementation.

Effective stakeholder engagement in the TrACER Study can be attributed to numerous factors. According to the results from the annual satisfaction survey completed in 2016 and 2017, ESAG members were satisfied with the methods, frequency and target areas of their engagement in the trial during project years 1 and 2. Across both years, ESAG members favored regular updates on recruitment and enrollment, as well as participation in discussions with the research team about implementation challenges and ways to troubleshoot issues in the trial. They also preferred practical, relevant communication about time-sensitive issues so that they could be “nimble and pivot as necessary” as one ESAG member said. Interestingly, ESAG members ranked in-person meetings as the most effective method for receiving information and providing input during project year 2, whereas they ranked in-person meetings as the least effective option in project year 1. However, receiving information via webinar was consistently a preferred option during both project years.

**Fig. 3:**
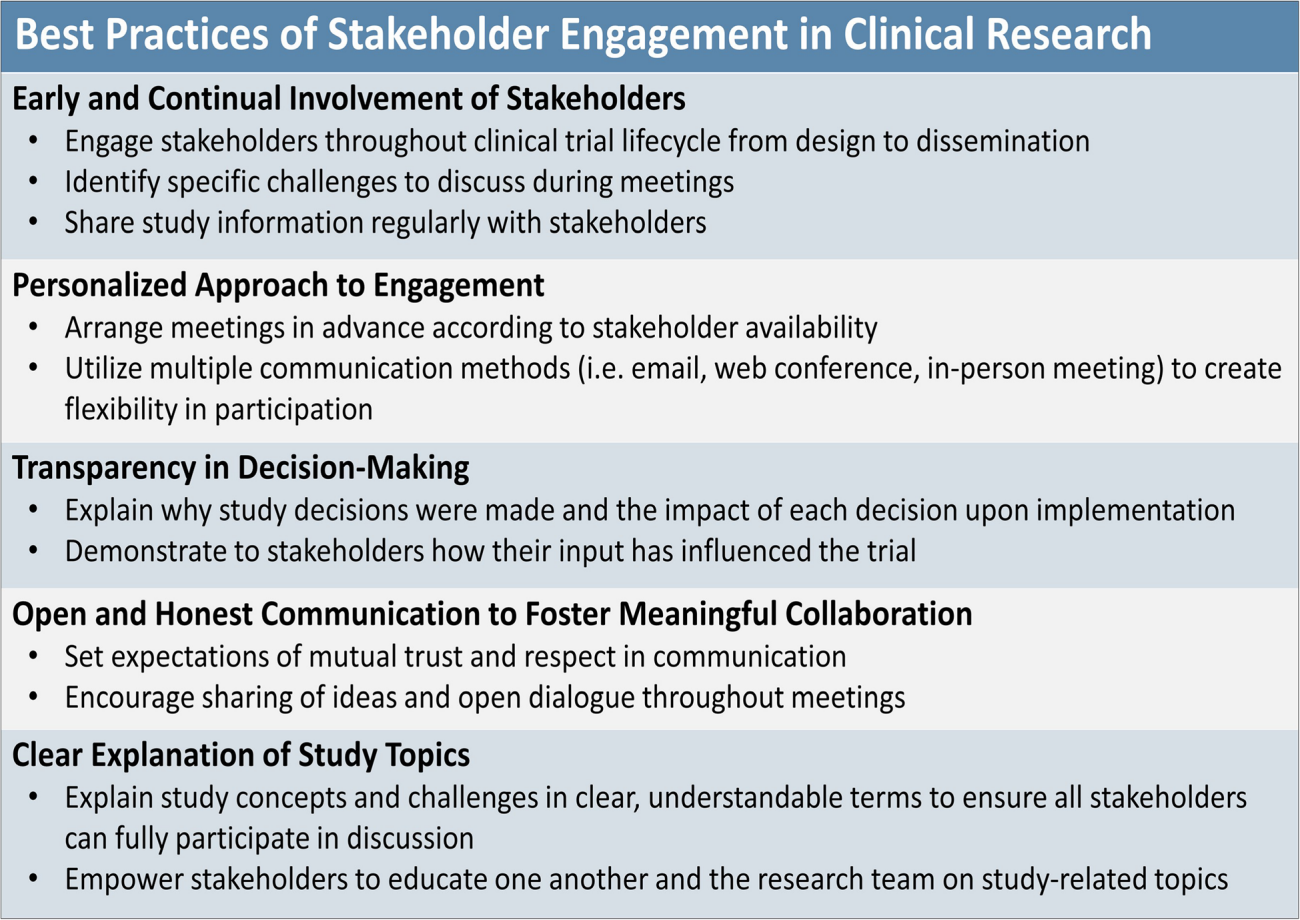
Best Practices of Stakeholder Engagement (H: 3.88″, W: 6″)

During year 3, ESAG members and the research team compiled a list of best practices of stakeholder engagement (Fig. 3) to help guide other research groups interested in incorporating stakeholder engagement into health care delivery research and clinical trials.

## Conclusions

Effective stakeholder engagement in the design, conduct and reporting of clinical research is essential to project success, particularly in cancer care delivery research. We have described the approach by which we engaged a diverse set of stakeholders for a pragmatic clinical trial testing a method to improve the appropriate use of an expensive supportive therapy for cancer. Our experience illustrates how different perspectives can lead to improved, patient-centric design and facilitate implementation of a trial. The lessons described in the manuscript may assist others with interest in effective use of stakeholders to inform clinical research.

## Data Availability

Not applicable.

## Abbreviations

ASCO: American Society of Clinical Oncology
CER: Comparative effectiveness research
CSF: Colony stimulating factors
ESAG: External Stakeholder Advisory Group
FN: Febrile neutropenia
NCCN: National Comprehensive Cancer Network
NCORP: National Cancer Institute Community Oncology Research Program
PCOR: Patient-centered outcomes research
PCORI: Patient-Centered Outcomes Research Institute
SWOG: Formerly the “Southwest Oncology Group,” now called SWOG Cancer Research Network
TrACER: Trial Assessing CSF Prescribing Effectiveness and Risk
